# Incidence Assessment of MTHFR C677T and A1298C Polymorphisms in Iranian Non-syndromic Cleft Lip and/or Palate Patients

**DOI:** 10.15171/joddd.2015.020

**Published:** 2015-06-10

**Authors:** Asghar Ebadifar, Nazila Ameli, Hamid Reza Khorramkhorshid, Mehdi Salehi Zeinabadi4, Kourosh Kamali, Tayyebeh Khoshbakht

**Affiliations:** ^1^Associate Professor, Dentofacial Deformities Research Center, Research Institute of Dental Sciences, Shahid Behehsti University of Medical Sciences, Tehran, Iran; ^2^Assistant Professor of Orthodontics, Dental School, Semnan University of Medical Sciences, Semnan, Iran; ^3^Professor, Genetic Research Centre, University of Social Welfare and Rehabilitation Sciences, Tehran, Iran; ^4^Assistant Professor of Pediatric Dentistry, Dental School, Semnan university of Medical Sciences, Semnan, Iran; ^5^Reproductive Biotechnology Research Center, Avicenna Research Institute, ACECR, Tehran, Iran; ^6^MSC, Genetic Research Centre, University of Social Welfare and Rehabilitation Sciences, Tehran, Iran

**Keywords:** A1298C, C677T, methylenetetrahydrofolate reductase, orofacial cleft, polymorphism.

## Abstract

***Background and aims.*** The aim of the present study is to determine the incidence of *MTHFR* C677 T and A1298C muta-tions in Iranian patients with cleft lip and/or cleft palate.

***Materials and methods.*** We screened 61 Iranian patients with cleft lip and/or cleft palate for mutations in the two alleles of *MTHFR* gene associated with cleft lip and/or palate: A1298C and C677T, using Polymerase Chain Reaction following by RFLP.

***Results.*** The 677T and 1298C homozygote genotypes showed a frequency of 36.1% and 11.4%, respectively. Combined genotype frequencies in newborns having oral clefts showed that the highest genotype was 677TT/1298AA (22.9%) and 677TT/1298CC genotypes were not observed.

***Conclusion.*** The results showed that 65.6% of all patients had at least one T mutant allele in C677T and 58.9% C mutant allele for A1298C. According to the frequencies of homozygosity of mutant alleles, it could be said that *MTHFR* genotype of 677TT shows a greater role in having oral clefts.

## Introduction

Cleft lip with or without cleft palate (CL/P) is one the most common orofacial congenital defects for which many epidemiological studies have assessed the prevalence.^1-6^Non-syndromic CL/P is believed to have a multifactorial threshold inheritance genetic model.^7^

Evidence shows that variations in genes associated with folate metabolism may greatly take part in the etiology of non-syndromic CL/P. Among genes related to folate metabolism, the methyletetrahydrofolate reductase gene (*MTHFR*) has been shown to be the most frequent one associated with non-syndromic CL/P. This gene translates to an enzyme which catalyzes the methylation of homocysteine amino acid to methionine. Defects in this pathway lead to metionine deficiency and the accumulation of homocysteine.

Despite the critical role of methionine as an important precursor in the DNA and RNA methylation process, high serum homocysteine levels are teratogenic during the embryogenesis.^8,9^ Two common polymorphisms (C→T and A→C) exist within the *MTHFR* gene at positions 677 and 1298, respectively.^10^ Results regarding the association of *MTHFR* gene polymorphism and the risk of nonsyndromic CL/P have been contradictory. None of the previous studies have examined Iranian patients. Therefore, the aim of this study was to assess the role of *MTHFR* C677T and A1298C in the development of non-syndromic CL/P in an Iranian population.

## Materials and methods

Sixty-one patients with CL/P from those who were referred to Mofid Hospital in Tehran, Iran, were selected for the purpose of this study. The selected patients had non-syndromic cleft lip and/or palate; those with other facial or skeletal malformations, metabolic or neurologic disorders or anomalies of other organs were excluded in case selection.

Ethical approval for the study was obtained from the Ethics Committee of the Dental Faculty of the Shahid Beheshti University of Medical Sciences. Written informed consents were obtained from all participants. In order to identify any possible prenatal contributory teratogenic factor that might have influenced the development of CL/P, a detailed questionnaire was completed. The questionnaire was modeled on the United States Centers for Disease Control (CDC) questionnaire for risk factor surveillance for birth defects. We asked all mothers for maternal illnesses, medication intake, history of abortion, history of cardiovascular disease, smoking and amount of folate intake during the periconceptional period (ranging from 3 months prior to 1 month after conception).

Blood samples were collected using a sterile 2-ml disposable syringe. Blood samples were collected in tubes containing 200 µl 0.5-M EDTA and stored at −80°C until further analysis. Genotyping for C677T and A1298C *MTHFR* gene mutations was performed by enzymatic restriction digestion of PCR products with HinfI and MboII (Fermentas, Germany), respectively.^11,12^

For screening of the 677C→T and 1298A→C variants in the *MTHFR* gene, exon 4 and 7 of the gene were amplified by polymerase chain reaction (PCR) following standard conditions and with the use of modified primers: (4F:5'-TCTTCATCCCTCGCCTTGAAC-3'; 4R:5'-AGGACGGTGCGGTGAGAGTG-3' and 7F:5'-CTTCTACCTGAAGAGCAAGTC-3' 7R:5'-CATGTCCACAGCATGGAG-3', respectively). DNA fragments were separated and visualized by electrophoresis using 2% agarose gels.

## Results

Sixty-one subjects were available for analysis. All patients had Iranian background.

Tables 1 & 2show the prevalence of different genotypes for the C677T and A1298C polymorphisms. The frequency of homozygosity for mutant T allele in C677T is much greater than for mutant C allele in A1298C (almost three folds).

**Table 1:** Genotype frequency of *MTHFR* C677T

	**genotype**	**CC**	**CT**	**TT**
**Count**		21	18	22
**% Within group**		34.4%	29.5%	36.1%

**Table 2:** Genotype frequency of *MTHFR* A1298C

	**genotype**	**AA**	**AC**	**CC**
**Count**		25	29	7
**% Within group**		40.9%	47.5%	11.4%

Homozygosity for the C677T *MTHFR* SNP was detected in 36.1%. The frequency of the C677T heterozygotes was 29.5%. Also, homozygosity for the *MTHFR* A1298C SNP was detected in 11.4% of the tested individuals. 47.5% were heterozygote for this SNP.

Table 3 shows the prevalence of combined C667T/A1298C genotype frequencies. The highest prevalence was represented by the 677TT/1298AA genotype combination‏ (22.9%), while the 677TT/1298CC genotype was not observed.

**Table 3:** Prevalence of combined genotypes

***MTHFR***	**C677C N (%)**	**C677T N (%)**	**T677T N(%)**
**A1298A**	5 (8%)	6 (9.8%)	14 (22.9%)
**A1298C**	12 (19%)	9 (14.7%)	8 (13.1%)
**C1298C**	4 (6.5%)	3 (4.9%)	0

## Discussion

Over the last few years, impaired *MTHFR* has been widely investigated in order to establish its potential role as a risk factor or marker of cardiovascular disease, neural tube defects, cognitive disorders, and cancer,^13,14^ despite the frequently conflicting literature.

The *MTHFR* C677T transition induces an enzyme theromlability with an *in-vitro* reduced enzyme activity ranging from 30-70% in hetero and homozygotes, respectively.^15^ The *MTHFR* A1298C polymorphism has been less investigated; the 1298C allele is responsible for reduced enzyme activity although it is less deteriorating than the 677T allele. The phenotypic expression of these mutations, especially for the 677T polymorphism can be conditioned by environmental interactions. In this context, folate is of great importance because an inadequate folate intake in addition to reduced *MTHFR* activity could facilitate the clinical expression of the *MTHFR* mutation-disease association.^16^

The genotype prevalence and allele frequencies of the two polymorphisms have not been reported in CL/P patients previously. In a study conducted by Yanamandra et al,^17^ heterozygote (CT) and variant homozygote (TT) frequencies of 677TC were 18%– 46%–45% and 0.9%–21%–11%, respectively, for African-American–Hispanics–Caucasians. Furthermore, 677TT variant homozygote in Hispanic newborn population was higher than other ethnic groups which can explain the higher prevalence of neural tube defects in this population.^17^ Another study in Italy showed the prevalence of 677TT and 1298CC genotypes was 25% and 47.1%, respectively.^18^

To date, only a few studies have evaluated the A1298C genotype prevalence all over the world. Thus, the results must be confirmed by further larger epidemiologic studies. Several studies describe the absence of other combined genotypes such as the 677CT/1298CC and 677TT/1298AC, which we found at low percentages (4.9% and 13.1%, respectively).

Despite the concern for double heterozygosity of the 677T and 1298C allele, our results are not in agreement with previous studies. Although this genotype combination has been proposed to predispose a reduction in *MTHFR* activity of about 50% and an increase in homocysteine level, the data do not relate to children with oral clefts. Therefore, it could be claimed that TT genotype is an important factor in developing oral clefts, while polymorphism in 1298AC does not have a critical role.^13,14,1^

The present investigation is among the few studies in this regard that have analyzed the prevalence of *MHTFR* genotypes in a newborn population.^20,21^ Moreover, the present study evaluated the prevalence of the two *MTHFR* polymorphisms in an Iranian population with CL/P.

The close relationship between *MTHFR* polymorphisms, folate serum levels in mothers, and the risk of orofacial clefts in children raises the question about the use of dietary supplements containing folic acid by pregnant women. The study of the prevalence of these polymorphisms in different geographical areas could be of interest in evaluating the possible clinical application deriving from the preventive folate supplementation.

## Acknowledgments

The authors would like to thank Dr. Roozrokh, Head of Mofid Hospital, the staff at the hospital, and the Genetic Research Center for their kind help in recruiting study subjects. This article was derived from the research performed by Dr. Nazila Ameli for her Post-graduate Degree in Orthodontics under the supervision of Dr. Ebadifar and supported by grants from Dentofacial Deformities Research Center, and Research Institute of Dental Sciences, both at Shahid Behehsti University of Medical Sciences, Tehran, Iran.

## References

[1] Deepak C, Venkatesan A, Ramanathan A (2012). In search of mutations of MSX1 gene in Indian nonsyndromic cleft palate patients. J Ind Orthod Soc.

[2] Derijcke A, Eerenes A, Carels C (1996). The incidence of oral clefts: a review. Br J Oral Maxillofac Surg.

[3] Das SK, Runnels RS Jr, Smith JC, Cohly HH (1995). Epidemiology of cleft lip and cleft palate in Mississippi. South Med J.

[4] Robert E, Kallen B, Harris J (1996). The epidemiology of orofacial clefts. 1. Some general epidemiological characteristics. J Craniofac Genet Dev Biol.

[5] Taher AA (1992). Cleft lip and palate in Tehran. Cleft Palate Craniofac J.

[6] Rajabian MH, Sherkat M (2000). An epidemiologic study of oral clefts in Iran: analysis of 1669 cases. Cleft Palate Cra-niofac J.

[7] Carter CO (1969). Genetics of common disorders. Br Med Bul.

[8] Limpach A, Dalton M, Miles R, Gadson P (2000). Homocysteine inhibits retinoic acid synthesis: a mechanism for homocysteine- induced congenital defects. Exp Cell Res.

[9] Greene ND, Dunlevy LE, Copp AJ (2003). Homocysteine is embryotoxic but does not cause neural tube defects in mouse embryos. Anat Embryol (Berl).

[10] Reutter H, Birnbaum S, Lacava AD, Mende M, Henschke H, Berge S (2008). Family-based association study of the MTHFR polymorphism C677T in patients with nonsyndromic cleft lip and palate from Central Europe. Cleft Palate Craniofac J.

[11] van der Put NM, Steegers-Theunissen RP, Frosst P, Trijbels FJ, Eskes TK, van den Heuvel LP (1995). Mutataed methylenetetrahydrofolate reductase as a risk factor for spina bifida. Lancet.

[12] van der Put NM, Gabreels F, Stevens EM, Smeitink JA, Trijbels FJ, Eskes TK (1998). A second common mutation in the methylenetetrahydrofolate reductase gene: an additional risk factor for neural tube defects?. Am J Hum Genet.

[13] Boccia S, Hung R, Ricciardi G, Gianfagna F, Ebert MP, Fang JY (2008). Meta- and pooled analyses of the methylenetetrahydrofolate reductase C677T and A1298C polymorphisms and gastric cancer risk: a huge-GSEC review. Epidemiol.

[14] D'Uva M, Di Micco P, Strina I, Alviggi C, Iannuzzo M, Ranieri A (2007). Hyperhomocysteinemia in women with unexplained sterility or recurrent early pregnancy loss from Southern Italy: a preliminary report. Thromb J.

[15] Frosst P, Blom HJ, Milos R, Goyette P, Sheppard CA, Matthews RG (1995). A candidate genetic risk factor for vascular disease: A common mutation in methylenetetrahydrofolate reductase. Nat Genet.

[16] Kang SS, Wong PWK (1996). Genetic and nongenetic factors for moderate hyperhomocysteinemia. Atherosclerosis.

[17] Yanamandra K, Naper D, Jalanivich DW, Thurmon TF (2000). Prevalence of methylenetetrahydrofolate reductase (MTHFR) C677T polymorphism in Northwest Louisiana newborn population. Genet Med.

[18] Zappacosta B, Romano L, Persichilli S, Cutrone LA, Graziano M, Vitrani A (2009). Genotype prevalence and allele frequencies of 5,10 methylenetetrahydrofolate reductase (MTHFR) C677T and A1298C polymorphisms in Italian newborns. Lab Medicine.

[19] Verkleij-Hagoort A, Bliek J, Sayed-Tabatabaei F, Ursem N, Steegers E, Steegers-Theunissen R (2007). Hyperhomocysteinemia and MTHFR polymorphisms in association with orofacial clefts and congenital heart defects: a meta-analysis. Am J Med Genet A.

[20] Wilcken B, Bamforth F, Li Z, Zhu H, Ritvanen A, Renlund M (2003). Geographical and ethnic variation of the 677C>T allele of 5,10 methylenetetrahydrofolate reductase (MTHFR): findings from over 7000 newborns from 16 areas world wide. J Med Genet.

[21] Isotalo PA, Wells GA, Donnelly JG (2000). Neonatal and fetal me-thylenetetrahydrofolate reductase genetic polymorphism: an examination of C677T and A1298C mutations. Am J Hum Genet.

